# Effects of green tea based oral health strategies on disease activity in rheumatoid arthritis

**DOI:** 10.3389/fmed.2024.1413753

**Published:** 2024-11-05

**Authors:** Sanrong Lan, Shuang Jin, Rong Lin, Baochuan Chen, Fei Gao, Shengli Zhang, Lili Chen, Hong Li

**Affiliations:** ^1^Shengli Clinical Medical College of Fujian Medical University, Fuzhou, China; ^2^The School of Nursing, Fujian Medical University, Fuzhou, China; ^3^Fujian Provincial Hospital, Fuzhou, China; ^4^Fujian Medical University 2nd Affiliated Hospital, Quanzhou, China

**Keywords:** rheumatoid arthritis, periodontitis, self-health management, oral care, self-care ability

## Abstract

**Aim:**

In this study, we aimed to evaluate oral health strategies in rheumatoid arthritis (RA) patients with periodontitis.

**Methods:**

We enrolled 110 RA patients with periodontitis who were diagnosed in a Grade A tertiary hospital into an oral health strategies program. The control and test groups comprised 55 cases each. The management effect was evaluated by self-care ability, oral health-related quality of life, RA-related clinical indicators, and the DAS28 score. The control group received routine nursing, whereas the test group was in a self-health management program for 3 months.

**Results:**

After the intervention, compared to the control group, the test group showed better self-care ability, oral health-related quality of life score, RA-related clinical indicators, and DAS28 score (*P* < 0.05).

**Conclusion:**

Our oral health strategies program slowed down the progression of the disease and can be popularized in patients.

## 1 Introduction

Rheumatoid arthritis (RA), a common autoimmune disease (1), is characterized by chronic aggressive arthritis (2) and a high disability rate (3). If it is not treated as per the standardized protocols, it can eventually lead to joint deformity and loss of function (4). Its pathophysiology and disease control are closely related to oral microbial infection. The prevalence of periodontitis is higher in patients with RA than in the normal population (5), and the presentation of the disease is more severe as well (6). Periodontitis is a chronic, infectious periodontal disease wherein microorganisms on the surface of the tooth (mainly gram-negative anaerobic bacteria) destroy periodontal support tissues and gradually form periodontal pockets, which in turn cause loss of clinical attachment and alveolar bone resorption (7). Both RA and periodontitis can destroy surrounding soft tissue and bone structure (8), and studies have shown that the pathogenesis of RA and periodontitis is similar, with both affecting each other (9–11). Their pathogenesis is also related in many ways, such as epidemiology, loci, the bacteria involved, and immune and inflammatory factors (12, 13). Periodontitis is a risk factor for RA, and inflammation in RA can be significantly alleviated by effective treatment of periodontitis, which is aimed at controlling bacterial plaque and reducing periodontal pathogens (14). Periodontal treatment is beneficial for the control of clinical symptoms of RA and the reduction of laboratory indicators (15). Oral care intervention is an important part of standardized periodontal basic treatment of patients with RA (16). It is an important way to keep the mouth clean and control plaque and an important means to prevent the occurrence and slow down the development of periodontitis (17), which directly translates into a better oral health of patients (18). However, traditional oral care for patients with RA has many shortcomings. These patients may have limited movement of their hands and joints due to the illness, preventing them from maintaining proper oral hygiene (19). This results in a large accumulation of oral plaque and increases the risk of periodontitis. Consequently, patients are likely to develop and exhibit negative emotions like helplessness, irritability, and anxiety due to periodontal discomfort, and physical and psychological discomfort leads to reduced food and water intake (20).

Green tea contains tea polyphenols, which have antibacterial, anti-inflammatory, and anti-oxidative properties, among other biological activities. They can inhibit the growth of various bacteria. Therefore, green tea-based oral care strategies may help improve oral health and control disease in RA patients. It is recommended that RA patients undergo regular oral examinations and care to maintain a clean and healthy mouth. In addition, drinking green tea may also help relieve RA symptoms. This study aims to explore the use of green tea oral care solutions for oral cleaning, to guide patients on proper oral care practices and oral health maintenance, and to slow down the progression of RA disease activity.

## 2 Materials and methods

### 2.1 Design and randomization

Random number series were generated using EXCEL by clinical epidemiologists who were not involved in this study. Random numbers ranging from 1 to 110 were put into sealed, light-tight envelopes and kept safe. The person who performed this randomization was not familiar with this subject. After enrolling the patients into the study, a random sequence was generated using a table of random numbers. The numbers were ordered from small to large; those numbered 1–55 were assigned to the intervention group, and those numbered 56–110 were assigned to the control group. Each of the random numbers was assigned using non-transparent sealed envelopes. Individuals responsible for sample collection and data analysis were blinded to group assignment.

This study was approved by the Ethics Committee of Fujian Provincial Hospital (ethics approval number: K2020-08-006). It was performed in strict adherence with moral principles and with a rigorous scientific research attitude. It followed the principle of “voluntariness, confidentiality, and harmlessness” and ensured all patients' willingness to participate in this study.

### 2.2 Participants selection

This study enrolled RA patients with periodontitis who were hospitalized in the Department of Rheumatology and Immunology of Fujian Provincial Hospital between December 2019 and December 2020. Only those who met the following inclusion and exclusion criteria were selected. The inclusion criteria were as follows: (1) patients who were diagnosed as having RA by a rheumatologist and an immunologist according to the RA diagnostic criteria of ACR/EULAR in 2010 (21) in a tertiary A hospital and who were also diagnosed as having periodontitis as per the criteria for periodontitis detailed in “Periodontology” published in 2012 (22); (2) patients who agreed to receive an oral periodontal examination and could cooperate with completing the questionnaire survey and providing written informed consent; (3) patients with no fewer than two functional teeth in at least one 1/6 quadrant; (4) patients who could complete the examination on time and appear for timely follow-up visits to cooperate with this study; and (5) patients aged 18–75 years old. The exclusion criteria were as follows: (1) patients having other inflammatory diseases or systemic diseases (e.g., Sjogren's syndrome, systemic lupus erythematosus, and allergic purpura); (2) pregnant and lactating women, patients with disabilities, and patients with diabetes, cardiovascular disease, liver cirrhosis, or mental illness;(3) patients having had periodontal treatment within the last 3 months; and (4) edentulous patients.

### 2.3 Sample size calculation

According to the results of Chen et al. (23), it was estimated that each group needed 38 participants, and assuming a dropout rate of 20%, we concluded that the sample size needed was 46 participants per group. Therefore, 55 participants were recruited in each of the two groups (experimental and control groups).

### 2.4 Interventions

The control group received routine nursing, including admission education, disease-related knowledge introduction, medication guidance, and discharge education. The intervention received by the test group patients was oral health strategies, which included the following: (1) Baseline data collection was performed on the first day of admission with “one-on-one” on-site guidance. (2) Theoretical knowledge training was given on the third day of admission (training duration: 20–30 min), which was administered in the form of individual missionary education, group education, and group discussions. (3) Training was given on oral care skills on the 4th day of admission (training duration: 40–45 min), and in this training, an oral skills operation video was shown to the patients using a projector. The investigators then combined the oral model to demonstrate the basics of the Bass brushing teeth technique, the use of dental floss sticks, and the use of the right green tea mouthwash. Patients were encouraged to give feedback on the spot and were sent the oral skill operation video course package over WeChat so they could rewatch it if they wanted. (4) Daily dietary guidance training was held on the morning of the fifth day after admission (training duration: 10–20 min), and it was administered as on-site guidance, individual missionary education, and group education. Finally, (5) timely follow-up visits were conducted after discharge. Specific intervention measures are listed in Table 1.

**Table 1:** Oral health strategies intervention.

**Name**	**Intervention**
Baseline data assessment	The general information questionnaire was used to obtain the patients' basic information (e.g., sex, age, education, payment, smoking, drinking, the use of biologics, and smoking) and understand their oral health, and the self-care ability scale was used to evaluate the patient's ability to take care of their health needs.
Theoretical knowledge training	According to the evaluation findings, the knowledge needed for the patient's health is summarized, and the patient is guided to learn health-promoting behaviors, understand self-concept, establish a sense of responsibility for self-care, cultivate a healthy psychological state, and improve social support.
Bass tooth brushing	The nurse instructed the patient to make a 45° angle between the bristles of the toothbrush and the long axis of the teeth, point the bristles to the root of the tooth, brush 2–3 teeth at a time, brush the teeth 4–6 times in a short distance horizontally, and then move on to the next group of 2–3 teeth. A 3-min timer hourglass was handed out to control the brushing time accurately.
Using dental floss	Patients were instructed to cut into the gap between the two adjacent teeth, close to the tooth surface to form a “c” shape, move from the root to the crown, and repeat this process 4–6 times. It should be ensured that the force of brushing is restrained to avoid damage to the gums.
Mouthwash	Patients were instructed to gargle with green tea water after each meal. They were asked to alternately puff the cheeks and suck to ensure that no part of the mouth misses full contact with the mouthwash. This had to be performed for no <3 min each time, and 5 times a day (once in the morning, once before going to bed, and once after each of the three meals). For each wash, they were instructed to use 100 mL of green tea mouthwash and rinse the mouth 2–3 times repeatedly until it is clean.
Green tea water configuration method	The patients in the test group were given transparent water cups with measurement scales and provided ordinary green tea (20 g/bag) from the same manufacturer, with the production date within the last 1 year. To prepare the mouthwash, 20 g of ordinary green tea was brewed with 400 mL of hot water and cooled to ~35°C before use.
Daily diet guidance	Patients were instructed to not eat pungent, hot, or hard food items to avoid secondary damage to the periodontium. Patients were encouraged to eat more foods rich in protein and fiber, such as eggs, fish, lean meat, fresh vegetables, and fruits. Smokers were asked to quit smoking
Follow-up after discharge (every 2 weeks)	Members of the research group were sent to contact the patient every 2 weeks to inquire about the patient's physical condition and the implementation of oral health strategies at the time, to check the records of the patient's “Oral Hygiene Habit Action Record Form,” and to give feedback and suggest corrections in real time. Patients who earnestly completed the records were rewarded with small gifts. In addition, assistance was provided to patients to make follow-up appointments with their doctor.

### 2.5 Evaluation index

The evaluation index included general information and the findings on the Self-Care Competence Scale (ESCA) (24), Oral Health Impact Profile (the Oral Health Impact Profile, OHIP-14) (25), and DAS28 Scoring Form (26). For data collection, the paper questionnaire was used to collect data when the patients were admitted to the hospital and 3 months after the intervention. Oral health-related examinations were recorded by professionally trained periodontists before and 3 months after the intervention.

### 2.6 Statistical methods

EpiData version 3.1 (EpiData Software, Odense, Denmark) was used for data entry with the double-entry principle and to check for consistency. If any data inconsistencies were found, the original questionnaire responses were checked for confirmation in time. The data were statistically analyzed using IBM SPSS 25.0. Enumeration data were described by frequency and percentage and analyzed by the *X*^2^ test or Fisher's exact probability method. The measurement data were normally distributed, and the homogeneity of variance was described by mean ± standard deviation (χ ± s). Within-group comparisons were performed using the paired *t*-test, and the between-group comparisons were performed using two independent samples *t*-test; the measurement data were non-normally distributed. The measurement data were non-normally distributed and described as the median (25% quantile to 75% quantile). Nonparametric tests were used, with the Wilcoxon rank sum test used for intra-group comparisons and the Mann–Whitney *U*-test used for inter-group comparisons.

### 2.7 Safety

Throughout the study, no adverse events or deaths occurred in either group.

### 2.8 Sample loss instructions

**Figure 1:**
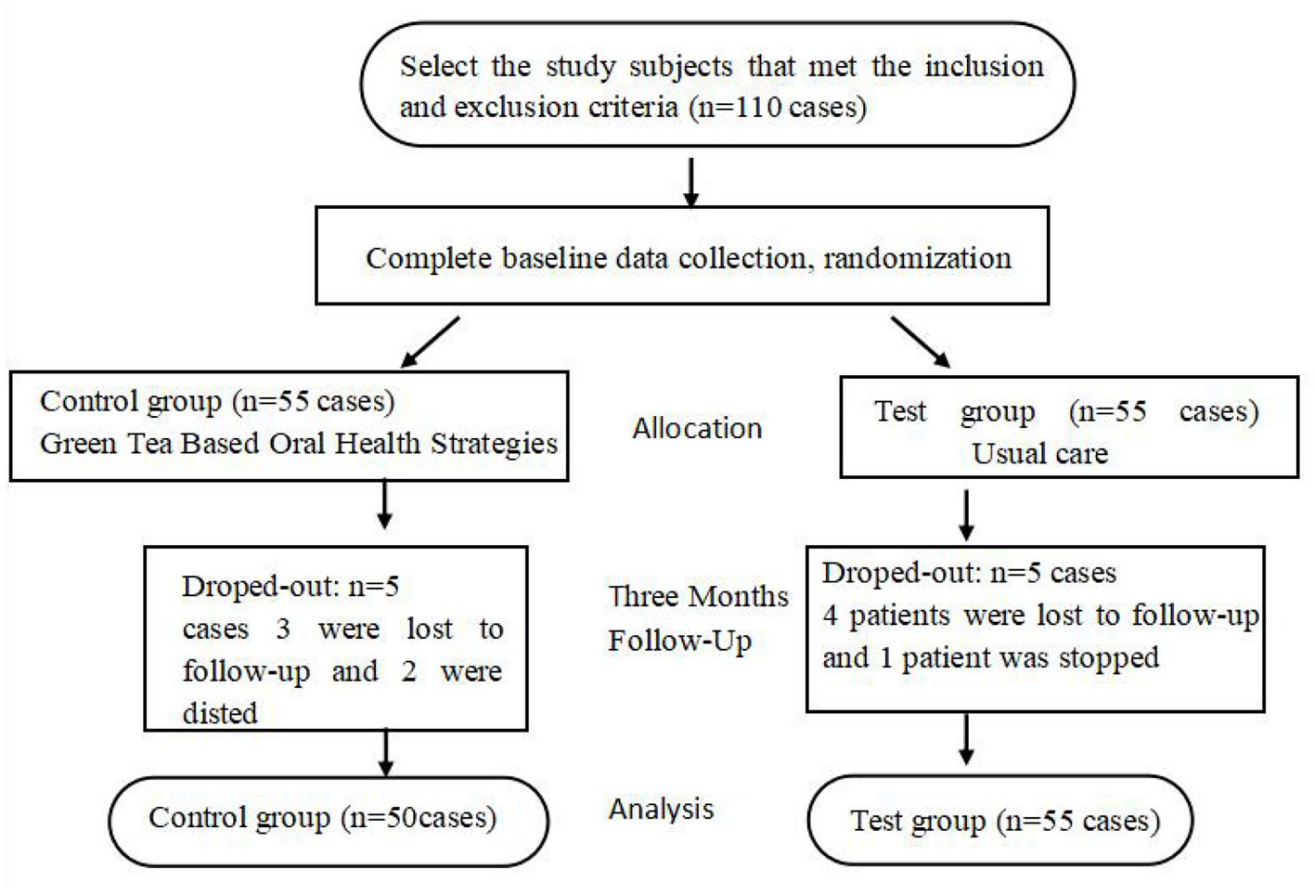
Sample loss instructions.

In test group four patients were lost to follow-up, and one patient was suspended due to change in disease condition; in control group three patients were lost to follow-up, and two patients were suspended due to change in disease condition. Finally, 100 study subjects completed all the data collection, as shown in Figure 1.

## 3 Results

In this study, 110 patients were included, including 55 patients in the experimental group and 55 patients in the control group.

In the experimental group, four patients were lost to follow-up, and one patient's enrollment was canceled due to the change of condition. In the control group, three patients were lost to follow-up, and two patients' enrollment was canceled due to the change of condition. Finally, 100 patients provided all the required data, with 50 patients in each group.

### 3.1 Comparison of the general data of the two patient groups

The general data of the two patient groups were comparable with no significant difference (*P* > 0.05; Table 2).

**Table 2:** Comparison of general data of two patient groups (cases, %).

**Projects**		**Test (*n* = 50)**	**Control (*n* = 50)**	***χ^2^***	***P***
Sex	Male	9 (18.00)	13 (26.00)	0.932	0.334
	Female	41 (82.00)	37 (74.00)		
Age (years)		57 (19–75)	58 (18–75)	2.785	0.248
Education	High school or below	47 (94.00)	43 (86.00)	1.778	0.182^a^
	High school above	3 (6.00)	7 (14.00)		
Payment	Self-funded	5 (10.00)	4 (8.00)	1.498	0.473^a^
	Urban resident medical insurance	45 (90.00)	45 (90.00)		
	Provincial or city medical insurance	0 (0.00)	1 (2.00)		
Smoking	No	46 (92.00)	45 (90.00)	0.122	0.727^a^
	Yes	4 (8.00)	5 (10.00)		
Drinking	No	45 (90.00)	46 (92.00)	0.122	0.727^a^
	Yes	5 (10.00)	4 (8.00)		
Using biologicals (etanercept/adalimumab)	Yes	9 (18.00)	10 (20.00)	0.065	0.799
	No	41 (92.00)	40 (80.00)		
Using glucocorticoid	Yes	41 (92.00)	40 (80.00)	0.065	0.799
	No	9 (18.00)	10 (20.00)		

^a^Indicates using Fisher's precision probability method.

Smoking: Smoke more than one cigarette a day.

Drinking: Drink more than 3 times a week and drink more than 10 ml a day.

### 3.2 The outcome indicators of the two patient groups before and after the intervention

After the intervention, patients' self-care ability, oral health-related quality of life, RA-related clinical indicators, DAS28 score, and other indicators were significantly higher in the test group than in the control group (*P* < 0.05; Table 3).

**Table 3:** Comparison of outcome indicators between the two groups before and after intervention.

**Project**	**Group (*n* = 50)**	**Before intervention**	**After intervention**	**Difference**	***t/Z***	***P***
Self-care score	Test	93.26 ± 10.68	110.82 ± 10.02	17.62 ± 7.22	−17.211	<0.001
	Control	90.88 ± 8.80	91.72 ± 7.26	0.84 ± 3.19	−1.862	0.690
	*t*	1.216	10.915	14.988		
	*P*	0.227	0.012	<0.001		
Oral health-related life quality score	Test	23.64 ± 4.31	16.12 ± 3.41	−7.00 (−10.00 to −4.00)^*^	12.916	<0.001
	Control	22.70 ± 3.17	22.36 ± 3.37	−1 (−2.25 to 2.00)^*^	0.647	0.521
	*t/Z*	1.243	−9.209	−7.349^a^		
	*P*	0.217	<0.001	<0.001		
Tender joints (number)	Test	12.08 ± 4.16	0.00 (0.00–1.00)^*^	−12.00 (−14.00 to −10.00)^*^	−6.156^a^	<0.001
	Control	12.20 ± 4.25	0.0 (0.00–1.00)^*^	−9.00 (−12.00 to −8.00)^*^	−6.025^a^	<0.001
	*t/Z*	−0.142	−7.448^a^	−2.783^a^		
Swollen joints (number)	Test	2.00 (2.00–3.25)^*^	0.00 (0.00–0.00)^*^	−2.00 (−3.00 to −1.00)^*^	−5.960^a^	<0.001
	Control	2.00 (2.00–4.00)^*^	1.00 (0.00–2.00)^*^	−2.00 (−2.00 to 0.00)^*^	−4.663^a^	<0.001
	*t/Z*	−0.790^a^	−4.715^a^	2.246^a^		
	*P*	0.430	<0.001	0.025		
ESR	Test	27.08 ± 5.45	17.32 ± 4.89	−9.00 (−14.00 to −5.50)^*^	9.443	<0.001
	Control	27.22 ± 5.72	25.10 ± 3.62	0.00 (−5.25 to 0.00)^*^	2.621	0.012
	*t/Z*	−0.125	−9.038	−5.364^a^		
	*P*	0.901	<0.001	<0.001		
DAS28 score	Test	5.13 ± 0.51	2.49 ± 0.40	−2.59 (−3.05 to −2.29)^*^	27.865	<0.001
	Control	5.20 ± 0.49	3.66 ± 0.40	−1.54 (−1.83 to −1.24)^*^	17.891	<0.001
	*t/Z*	0.655	−14.804	−7.142^a^		
	*P*	0.539	<0.001	<0.001		

^*^Indicates non-normal distribution, using M (P25-P75).

^a^Indicates using the Mann–Whitney *U-*test.

The statistical results are *Z-*values, and others are *t-*values.

## 4 Discussion

### 4.1 Oral health strategies is beneficial to improve the self-care ability of RA patients with periodontitis

Self-care ability is the ability to achieve self-care through purposeful learning (27), which can promote and maintain the development of individual physical and mental health (28). Joint dysfunction and the consequently decreased self-care ability in patients with RA likely affect their oral hygiene behavior (29), and the use of immunosuppressants and hormones increases their vulnerability to developing oral microbial infections, resulting in cumulative damage to oral health status. The prognosis of patients with RA is closely associated with their self-care behavior (30).

This study revealed improved self-care ability of patients in the test group after the intervention, upholding the ability of the training program to improve their self-care ability. After training, the patients in the test group became more self-dependent, showed better mental states, and could master more comprehensive systematic oral self-care skills and knowledge. This empowered them to actively participate in the whole process of disease diagnosis, treatment, nursing, and rehabilitation, and this active participation translated into better self-care ability, early body recovery, shorter hospitalization time, and early return to normal daily life.

### 4.2 Oral health strategies is positive to improve the periodontal health status and oral health-related quality of life in patients with RA and periodontitis

The pathogenesis of periodontitis is complex, and the course of treatment is long, making effective oral care absolutely essential (31). However, in the passive oral cleaning process, nurses traditionally use cotton balls to wipe the patient's mouth. This requires the patients to keep their mouth open for a long time, making them prone to muscle fatigue and often causing nausea, retching, and other types of discomfort. Furthermore, as the passive cotton ball wiping method lacks a mouthwash step, oral bacteria tend to remain lodged in the corners of the mouth, which greatly shortens the duration of fresh breath, thus often leading to bad mental states, such as low self-esteem and emotional tension, in patients.

In this study, after 3 months of intervention and follow-up, RA patients with periodontitis showed significantly improved periodontal health status, which is also attributable to the program conforming to the patients' daily work and rest habits. Furthermore, the training also enhanced patients' awareness of the disease prevention and treatment, thus increasing their understanding of periodontitis-related risk factors, promoting the development of scientific oral hygiene habits, and improving the efficacy of their oral cleaning process. Upon admission, a periodontal specialist performed thorough oral cleaning, descaling, and plaque removal. Good oral care skills, such as correct way of using the tooth brush, rational use of dental floss, and green tea mouthwash can effectively maintain the dental health of patients with RA, and this is conducive to repairing damaged periodontal tissue and effectively preventing disease recurrence. The tea polyphenols in the green tea mouthwash used in this study could effectively inhibit the growth of bacteria and reduce dentin sensitivity (32), and they are used as an adjunctive treatment for chronic inflammatory diseases (33).

Oral health is reflective of an individual's health status and quality of life to a certain extent (34). Notably, the effects and consequences of periodontal disease may not be limited to the oral cavity. There is an association between periodontal disease and RA (35). In a previous study, participants were more willing to receive oral health education from a rheumatologist or dentist (36). In patient-centered dental care, it is important to recognize the physical effects of oral diseases and the psychosocial barriers that develop because of disease-related parameters (37). In patients with RA, regular oral examinations are reportedly needed to improve periodontal health (38). The present study showed that the oral health-related quality of life of patients in the test group was markedly improved. Our program combines patients' self-care needs and self-care ability, thus improving upon the traditional oral care model and allowing patients to take control of their oral health. After implementing the oral health strategies, the patients were encouraged to improve their self-responsibility and oral hygiene status, thus reducing oral infections and improving their oral comfort and quality of life.

### 4.3 Oral health strategies is favorrable to control oral disease in RA patients' activity

RA and periodontitis can cause continuous pathological bone resorption and harm health (39). Previous oral ulcers were reported in 30% of the patients (40). Controlling local periodontal infection and inflammation can reduce the systemic inflammatory response, which in turn helps improve the activity of patients with RA (41). The key to ensuring proper oral care and managing RA is maintaining awareness about them (42). In this study, we used the DAS28 score, which is the clinically relevant index of RA. It is a comprehensive evaluation index of long-term clinical attention and has been widely used to measure the activity of patients with RA.

In the test group, RA-related clinical indicators and DAS28 scores were significantly lower after the intervention than they were before the intervention (*P* < 0.05). The patients in the control group received conventional drug treatment for the disease, and even in these patients, the clinical indicators of the disease improved compared with before the treatment. Between-group comparisons showed that the differences in RA-related clinical indicators and the DAS28 score before and after the intervention were significantly greater in the test group than in the control group (*P* < 0.05), indicating that intervention measures can improve patients' RA-related clinical indicators, thus reducing patients activity, which is consistent with a previous study (43).

The final sample size collected in this study (50 cases in test group and control group) was small. To improve the reliability of the study results, further studies with larger sample sizes are needed. In this study, only inpatients from the rheumatism immunology department of a tertiary hospital were selected. The feasibility and scientific nature of the results in different regions need to be further verified. The intervention time of this study is 3 months, and future studies should implement a longer intervention time to improve the reliability of their study results. Tea mouthwash cannot replace periodontal treatment and should be used only as an adjunctive tool to enhance its results.

## 5 Conclusion

Green Tea Based Oral Health Strategies for RA patients with periodontitis is beneficial to improve the oral self-care ability of these patients; furthermore, it is optimistic to improve their oral health-related quality of life, periodontal health indicators, and activity levels. This study provides a reference basis for clinical oral care interventions for RA patients with periodontitis and adds new information for clinical medical staff to conduct oral health strategies.

## Data Availability

The original contributions presented in the study are included in the article/supplementary material, further inquiries can be directed to the corresponding author.

## References

[1] Zlatkovic-Svenda M Rouse M Radak-Perovic M Stojanovic R Vujasinovic-Stupar N Lazovic-Popovic B. Adaptation and validation of the Rheumatoid Arthritis Quality of Life (RAQoL) questionnaire for use in Serbia. Rheumatol Int. (2017) 37:641–6. 10.1007/s00296-016-3586-0 27796523

[2] Huang J Fu X Chen X Li Z Huang Y Liang C. Promising therapeutic targets for treatment of rheumatoid arthritis. Front Immunol. (2021) 12:686155. 10.3389/fimmu.2021.686155 34305919 PMC8299711

[3] Smolen JS Aletaha D Barton A Burmester GR Emery P Firestein GS. Rheumatoid arthritis. Nat Rev Dis Primers. (2018) 4:18001. 10.1038/nrdp.2018.1 29417936

[4] Larice S Ghiggia A Di Tella M Romeo A Gasparetto E Fusaro E. Pain appraisal and quality of life in 108 outpatients with rheumatoid arthritis. Scand J Psychol. (2020) 61:271–80. 10.1111/sjop.12592 31674683

[5] Wen S Beltrán V Chaparro A Espinoza F Riedemann JP. Association between chronic periodontitis and rheumatoid arthritis. A systematic review. Rev Med Chil. (2019) 147:762–75. 10.4067/S0034-98872019000600762 31859830

[6] Wolff B Berger T Frese C Max R Blank N Lorenz HM. Oral status in patients with early rheumatoid arthritis: a prospective, case-control study. Rheumatology. (2014) 53:526–31. 10.1093/rheumatology/ket362 24273047

[7] Nakayama K. Porphyromonas gingivalis and related bacteria: from colonial pigmentation to the type IX secretion system and gliding motility. J Periodontal Res. (2015) 50:1–8. 10.1111/jre.12255 25546073 PMC4674972

[8] Zhang J Xu C Gao L Zhang D Li C Liu J. Influence of anti-rheumatic agents on the periodontal condition of patients with rheumatoid arthritis and periodontitis: a systematic review and meta-analysis. J Periodontal Res. (2021) 56:1099–115. 10.1111/jre.12925 34514591

[9] Leech MT Bartold PM. The association between rheumatoid arthritis and periodontitis. Best Pract Res Clin Rheumatol. (2015) 29:189–201. 10.1016/j.berh.2015.03.001 26362738

[10] Zaatout N. Presence of non-oral bacteria in the oral cavity. Arch Microbiol. (2021) 203:2747–60. 10.1007/s00203-021-02300-y 33791834 PMC8012020

[11] Kurgan S Fentoglu Ö Önder C Serdar M Eser F Tatakis DN. The effects of periodontal therapy on gingival crevicular fluid matrix metalloproteinase-8, interleukin-6 and prostaglandin E2 levels in patients with rheumatoid arthritis. J Periodontal Res. (2016) 51:586–95. 10.1111/jre.12337 26575440

[12] de Molon RS Rossa C Jr Thurlings RM Cirelli JA Koenders MI. Linkage of periodontitis and rheumatoid arthritis: current evidence and potential biological interactions. Int J Mol Sci. (2019) 20:4541. 10.3390/ijms20184541 31540277 PMC6769683

[13] Hussain SB Botelho J Machado V Zehra SA Mendes JJ Ciurtin C. Is there a bidirectional association between rheumatoid arthritis and periodontitis? A systematic review and meta-analysis. Semin Arthritis Rheum. (2020) 50:414–22. 10.1016/j.semarthrit.2020.01.009 32113837

[14] Zini A Mazor S Timm H Barker ML Grender JM Gerlach RW. Effects of an oral hygiene regimen on progression of gingivitis/early periodontitis: a randomized controlled trial. Can J Dent Hyg. (2021) 55:85–94. 34221032 PMC8219070

[15] Yang N-Y Wang C-Y Chyuan I-T Wu K-J Tu Y-K Chang C-W. Significant association of rheumatoid arthritis-related inflammatory markers with non-surgical periodontal therapy. J Formos Med Assoc. (2018) 117:1003–10. 10.1016/j.jfma.2017.11.006 29174174

[16] Juan CY Hsu CW Lu MC. Increased dental visits in patients with rheumatoid arthritis: a secondary cohort analysis of population based claims data. BMC Oral Health. (2022) 22:609. 10.1186/s12903-022-02661-w 36522732 PMC9753417

[17] Worthington HV MacDonald L Poklepovic Pericic T Sambunjak D Johnson TM Imai P. Home use of interdental cleaning devices, in addition to toothbrushing, for preventing and controlling periodontal diseases and dental caries. Cochr Database Syst Rev. (2019) 4:Cd012018. 10.1002/14651858.CD012018.pub2 30968949 PMC6953268

[18] Niesten D. Oral health care and oral health-related quality of life of frail and care-dependent older people. Ned Tijdschr Tandheelkd. (2017) 124:589–92. 10.5177/ntvt.2017.11.17168 29136049

[19] Monsarrat P Vergnes J-N Cantagrel A Algans N Cousty S Kémoun P. Effect of periodontal treatment on the clinical parameters of patients with rheumatoid arthritis: study protocol of the randomized, controlled ESPERA trial. Trials. (2013) 14:253. 10.1186/1745-6215-14-253 23945051 PMC3751435

[20] Rogers AA Willumsen T Strømme H Johnsen JK. Top-down self-regulation processes as determinants of oral hygiene self-care behaviour: a systematic scoping review. Clin Exp Dent Res. (2022) 8:807–26. 10.1002/cre2.548 35396799 PMC9382055

[21] Aletaha D Neogi T Silman AJ Funovits J Felson DT Bingham CO III. 2010 rheumatoid arthritis classification criteria: an American College of Rheumatology/European League Against Rheumatism collaborative initiative. Ann Rheum Dis. (2010) 69:1580–8. 20699241 10.1136/ard.2010.138461

[22] Meng HX. Periodontology. 4th ed. Beijing: People's Medical Publishing House (2012).

[23] Chen L Cao H Wu X Xu X Ji X Wang B. Effects of oral health intervention strategies on cognition and microbiota alterations in patients with mild Alzheimer's disease: a randomized controlled trial. Geriatr Nurs. (2022) 48:103–10. 10.1016/j.gerinurse.2022.09.005 36155316

[24] Kearney BY Fleischer BJ. Development of an instrument to measure exercise of self-care agency. Res Nurs Health. (1979) 2:25–34. 10.1002/nur.4770020105 254279

[25] Guo L Söderhamn U McCallum J Ding X Gao H Guo Q. Testing and comparing two self-care-related instruments among older Chinese adults. PLoS ONE. (2017) 12:e0182792. 10.1371/journal.pone.0182792 28792975 PMC5549914

[26] van Riel PL Renskers L. The Disease Activity Score (DAS) and the Disease Activity Score using 28 joint counts (DAS28) in the management of rheumatoid arthritis. Clin Exp Rheumatol. (2016) 34(5 Suppl. 101):S40–4. 27762189

[27] Xu X Han J Li Y Sun X Lin P Chen Y. Effects of orem's self-care model on the life quality of elderly patients with hip fractures. Pain Res Manag. (2020) 2020:5602683. 10.1155/2020/5602683 32566061 PMC7256682

[28] Patra C. Patient care, self-care. AMA J Ethics. (2021) 23:E428–429. 10.1001/amajethics.2021.428 34038353

[29] Minnock P McKee G Kelly A Carter SC Menzies V O'Sullivan D. Nursing sensitive outcomes in patients with rheumatoid arthritis: a systematic literature review. Int J Nurs Stud. (2018) 77:115–29. 10.1016/j.ijnurstu.2017.09.005 29080437

[30] Zuidema R van Dulmen S Nijhuis-van der Sanden M Meek I van den Ende C Fransen J. Efficacy of a web-based self-management enhancing program for patients with rheumatoid arthritis: explorative randomized controlled trial. J Med Internet Res. (2019) 21:e12463. 10.2196/12463 31038461 PMC6658318

[31] Kapila YL. Oral health's inextricable connection to systemic health: special populations bring to bear multimodal relationships and factors connecting periodontal disease to systemic diseases and conditions. Periodontol 2000. (2021) 87:11–6. 10.1111/prd.12398 34463994 PMC8457130

[32] Melok AL Lee LH Mohamed Yussof SA Chu T. Green tea polyphenol epigallocatechin-3-gallate-stearate inhibits the growth of *Streptococcus mutans*: a promising new approach in caries prevention. Dent J. (2018) 6:38. 10.3390/dj6030038 30082585 PMC6162448

[33] Lippert A Renner B. Herb-drug interaction in inflammatory diseases: review of phytomedicine and herbal supplements. J Clin Med. (2022) 11:1567. 10.3390/jcm11061567 35329893 PMC8951360

[34] Schmalz G Noack S Patschan S Patschan D Müller GA Rupprecht A. Disease activity, morning stiffness and missing teeth are associated with oral health-related quality of life in individuals with rheumatoid arthritis. Clin Oral Investig. (2020) 24:3559–66. 10.1007/s00784-020-03226-3 32025884

[35] Falcao A Bullón P. A review of the influence of periodontal treatment in systemic diseases. Periodontol 2000. (2019) 79:117–28. 10.1111/prd.12249 30892764

[36] Protudjer JLP Billedeau C Hurst K Schroth R Stavropoulou C Kelekis-Cholakis A. Oral health in rheumatoid arthritis: listening to patients. JDR Clin Trans Res. (2022) 7:127–34. 10.1177/23800844211012678 33949224

[37] Schmalz G Patschan S Patschan D Ziebolz D. Oral-health-related quality of life in adult patients with rheumatic diseases-a systematic review. J Clin Med. (2020) 9:1172. 10.3390/jcm9041172 32325846 PMC7231140

[38] Shrestha S Pradhan S Adhikari B. Prevalence of periodontitis among rheumatoid arthritis patients attending tertiary hospital in Nepal. J Nepal Health Res Counc. (2020) 17:543–7. 10.33314/jnhrc.v17i4.1841 32001864

[39] Silvestre FJ Silvestre-Rangil J Bagán L Bagán JV. Effect of nonsurgical periodontal treatment in patients with periodontitis and rheumatoid arthritis: a systematic review. Med Oral Patol Oral Cir Bucal. (2016) 21:e349–54. 10.4317/medoral.20974 26946202 PMC4867209

[40] Magdy E Ali S. Stratification of methotrexate-induced oral ulcers in rheumatoid arthritis patients. Spec Care Dentist. (2021) 41:367–71. 10.1111/scd.12575 33559176

[41] de Pablo P Serban S Lopez-Oliva I Rooney J Hill K Raza K. Outcomes of periodontal therapy in rheumatoid arthritis: the OPERA feasibility randomized trial. J Clin Periodontol. (2023) 50:295–306. 10.1111/jcpe.13756 36415901 PMC10946499

[42] Radwan-Oczko M Duś-Ilnicka I Richards P Thomsen AM Rasmussen C. Evaluation of oral health status and oral care of patients with rheumatoid arthritis. Int J Dent. (2020) 2020:8896766. 10.1155/2020/8896766 33178279 PMC7648699

[43] Posada-López A Botero JE Pineda-Tamayo RA Agudelo-Suárez AA. The effect of periodontal treatment on clinical and biological indicators, quality of life, and oral health in rheumatoid arthritis patients: a quasi-experimental study. Int J Environ Res Public Health. (2022) 19:1789. 10.3390/ijerph19031789 35162812 PMC8835021

